# Use of dermal-fat grafts in the post-oncological reconstructive surgery of atrophies in the zygomatic region: clinical evaluations in the patients undergone to previous radiation therapy

**DOI:** 10.1186/1746-160X-8-33

**Published:** 2012-12-05

**Authors:** Francesco Inchingolo, Marco Tatullo, Andrea Pacifici, Marco Gargari, Alessio D Inchingolo, Angelo M Inchingolo, Gianna Dipalma, Massimo Marrelli, Fabio M Abenavoli, Luciano Pacifici

**Affiliations:** 1 Department of Dental Sciences and Surgery, University of Bari, Bari, Italy; 2 Department of Medical Biochemistry, Medical Biology and Physics, University of Bari, Bari, Italy; 3 Department of “Head and Neck Diseases”, Hospital “Fatebenefratelli”, Rome, Italy; 4 Department of Maxillofacial Surgery, Calabrodental, Crotone, Italy; 5 School of Dentistry, University of Bari, Bari, Italy; 6 Department of Surgical, Reconstructive and Diagnostic Sciences, University of Milano, Milan, Italy; 7 Department of Stomatology and Maxillofacial Sciences, University of Rome "Sapienza", Rome, Italy; 8 Department of Clinical Sciences and Translational Medicine, University of Rome "Tor Vergata", Rome, Italy; 9 Tecnologica Research Institute, Regenerative Medicine Section, Street E. Fermi, Crotone, Italy

**Keywords:** Dermal-fat grafts, Oncological surgery, Plastic surgery

## Abstract

**Introduction:**

Grafting of autologous adipose tissue can be recommended in some cases of facial plastic surgery. Rhabdomyosarcoma is a type of cancer that can also affect the orbit. Enucleation of the eye can cause atrophy of the corresponding hemiface and decreased orbital growth.

**Case report:**

We report a case of a female patient with a medical history of surgical enucleation of the right eyeball, who had received rhabdomyosarcoma radiation therapy in her youth. The patient presented with a depression in the right zygomatic region. We took a dermal-fat flap from the abdominal region, which had been previously treated.

**Results:**

The surgical outcome, 48 hours, and much clearly 31 days after the surgery, revealed that the right zygomatic region had returned to its proper anatomical shape, although there were still signs of postoperative edema.

**Discussion:**

Very damaged tissues, like those exposed to radiation therapy, are generally not suitable for grafting of adipose tissue.

**Conclusions:**

In the described case, we achieved a technically and aesthetically satisfying result despite the patient's medical history involving several perplexities about the use of autologous dermal-fat tissues, because of prior radiation therapy exposure. The clinical case shows that even a region exposed to radiation therapy can be a valid receiving bed for dermal-fat grafting.

## Introduction

Grafting of autologous adipose tissue can be recommended in some cases of facial plastic surgery to correct the congenital and traumatic alterations. However, some authors have reported unpredictable results about both the resorption rate of adipose tissue grafts and the quantification of the consequent surgically increased volume loss [1,2].

Besides, many authors have studied the behavior of adipose grafts in the areas treated with radiation therapy; these studies agree in considering these areas as being unsuitable for grafting of autologous adipose tissue [1,2].

Rhabdomyosarcoma is a type of cancer that can also affect the orbit. It is not a common neoplasm and has an estimated annual incidence of approximately 4–5 new cases per million children below 15 years of age. There is a slight prevalence in males, with a male/female ratio of 1.5:1. Sarcomas of the soft tissues, the most frequent of which is rhabdomyosarcoma, represent 7% of all pediatric malignant tumors [3].

Although the 5-year survival rate of patients with orbital unilateral rhabdomyosarcoma is beyond 85%, the extent of eradication therapy and of the subsequent radiation therapy has a destructive impact on the treated area [4-6].

As a matter of fact, destructive surgery is not followed by rehabilitation. These patients often present with adipose tissue atrophy associated with deforming fibrotic conditions; these biological manifestations result from the radiations affecting that area [6,7].

Enucleation of the eye, especially in small children, can cause atrophy of the corresponding hemiface and decreased orbital growth, which create reluctantly accepted aesthetic conditions [6].

The aim of this work is to describe a new surgical approach to atrophies in the zygomatic region of patients with previous oncological surgery and ocular radiation therapy: we discuss about a rationale for the use of dermal-fat grafts.

This is a new technique which has never been described before in the literature and it is innovative compared to the traditional techniques. This technique has the advantage of a natural final result; moreover, we can rule out any possibility of rejection. When we perform the surgery on irradiated tissues, which are therefore more vulnerable, we have the possibility of using autologous tissue causing no local reaction and providing an adequate tissue thickness.

This technique represents an excellent opportunity for treatment of this atrophies, but the only disadvantage is the need to remove tissue from a different region of the body.

The alternative surgical procedures may be the mobilization of closeness flaps, or the microsurgery or the lipofilling: the first could create more scarring, so it is not the most aesthetic alternative; the second is a much more invasive surgical technique and it has a duration longer operating with a poor predictability of results; in the third case, a good result requires more surgical sessions to achieve a stable result.

## Case report

We report a case of a 32 years old female patient, clinically healthy, with a medical history of surgical enucleation of the right eyeball, who had received rhabdomyosarcoma radiation therapy in her youth.

**Figure 1:**
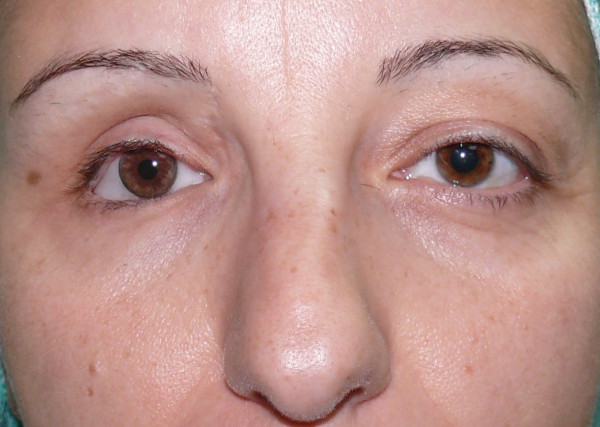
Pre-operative picture.

**Figure 2:**
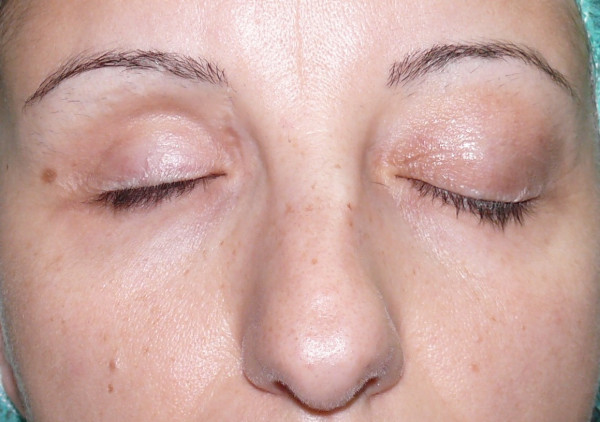
Pre-operative picture.

The patient presented with a depression in the right zygomatic region, upper eyelid asymmetry, and a slight right hemiface dimorphism, as compared to the left hemiface (Figures 1, 2).

Therefore, we decided to proceed with autologous tissue grafting in that region.

**Figure 3:**
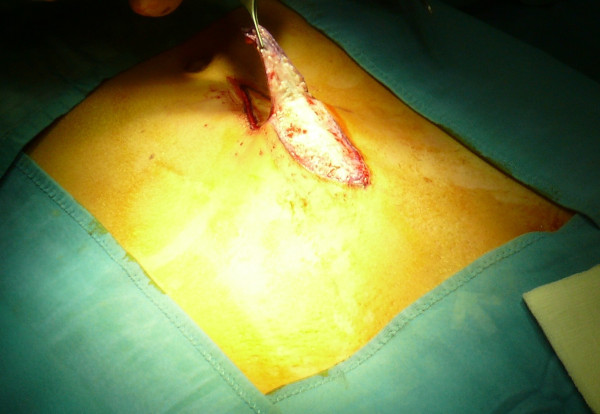
Incision in the sovrapubic region.

**Figure 4:**
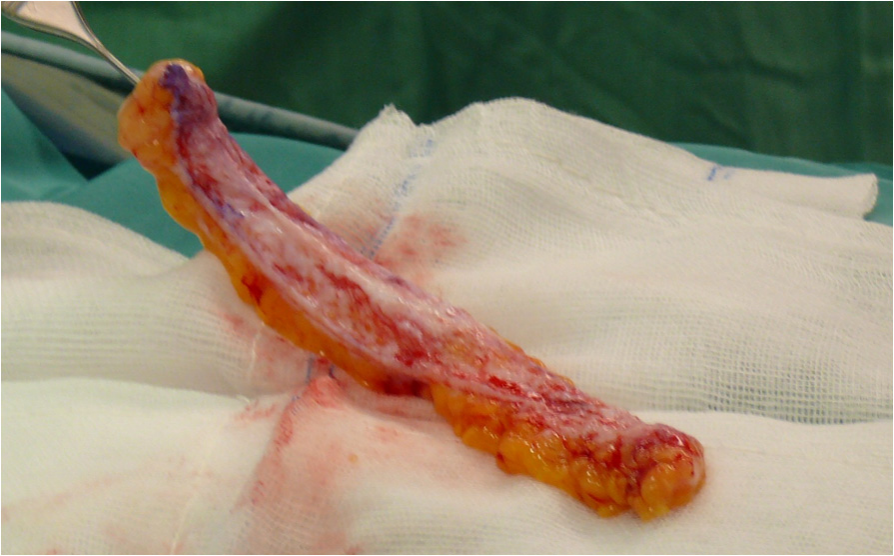
A dermal-fat flap taken from the abdominal region.

Subject to incision in the sovrapubic region, we took a dermal-fat flap from the abdominal region, which had been previously treated (Figures 3, 4).

**Figure 5:**
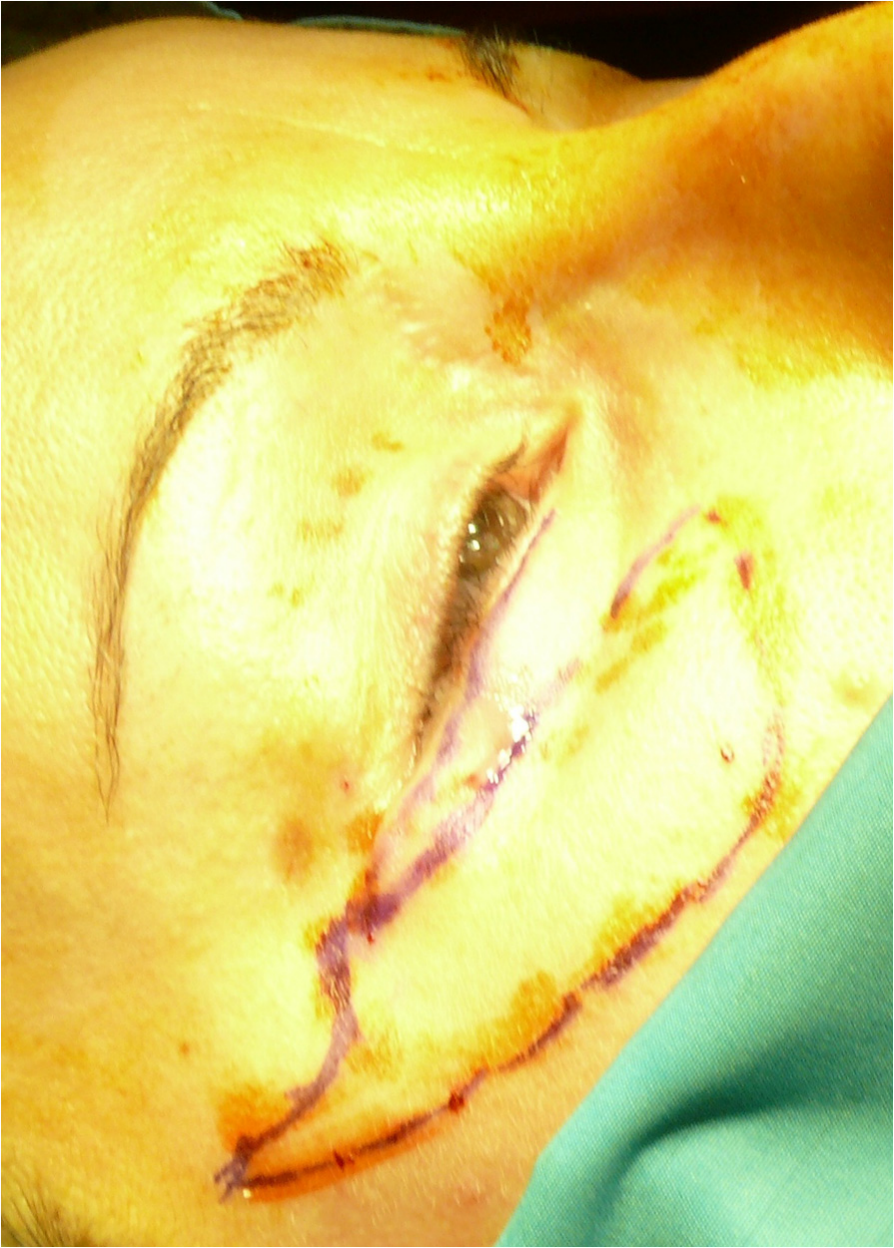
Design of the operative area.

**Figure 6:**
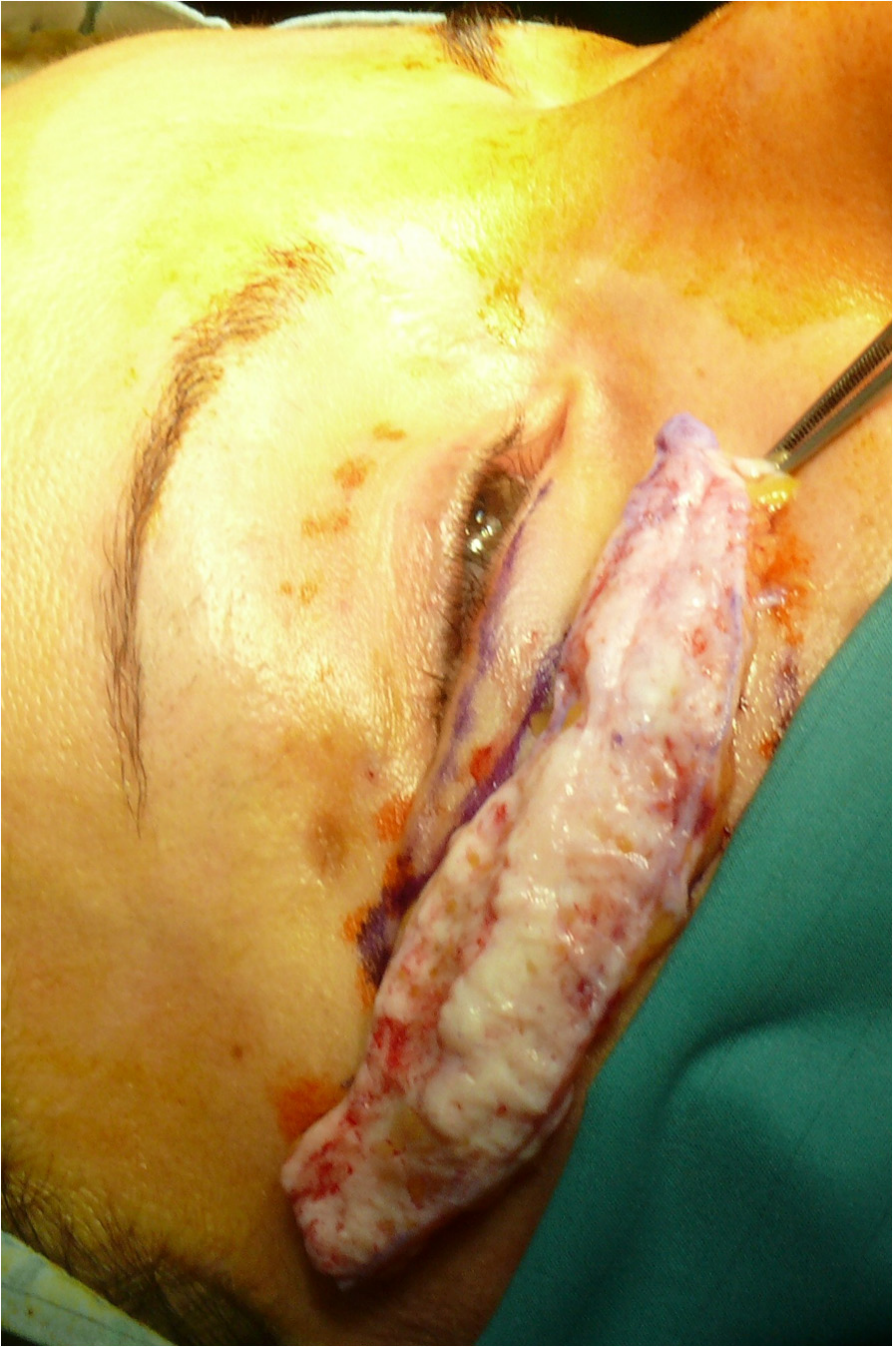
A slightly oversized dermal-fat flap.

**Figure 7:**
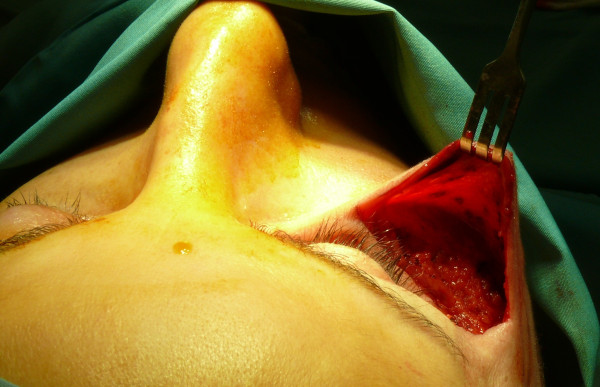
The bag detached around the right zygomatic region affected by tissue loss.

**Figure 8:**
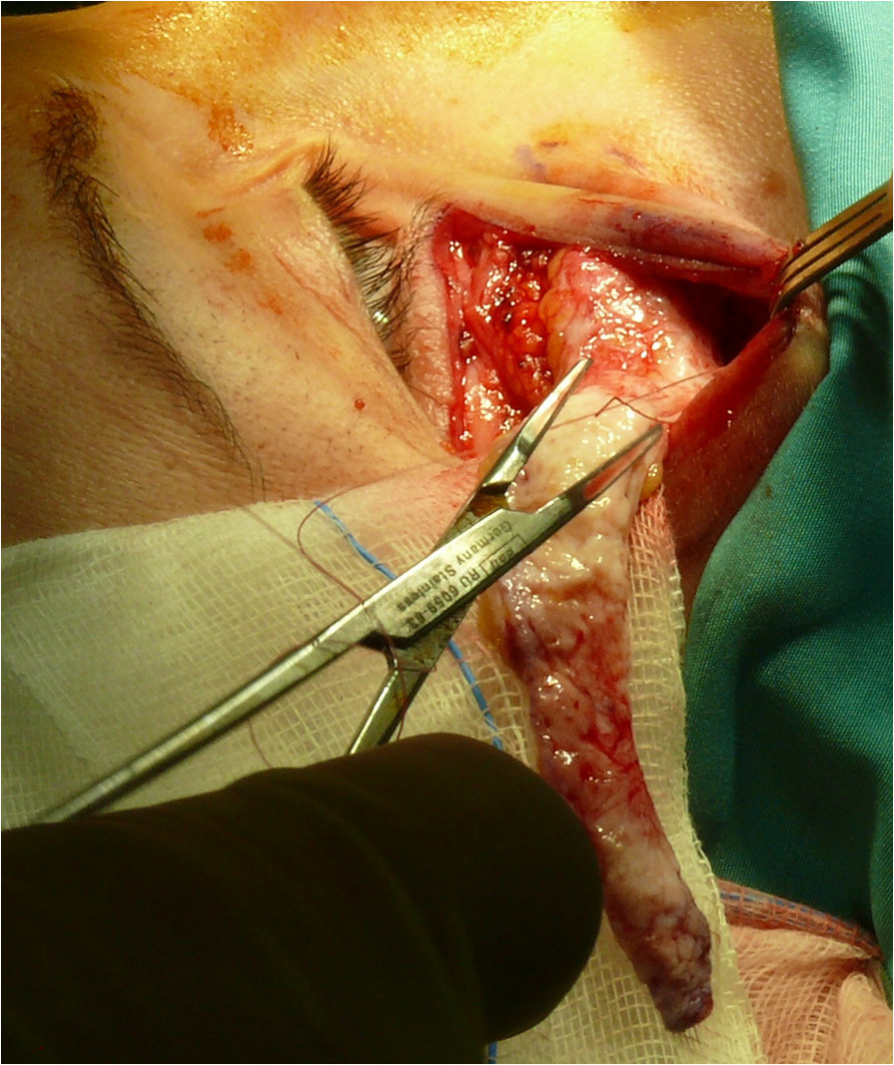
Dermal-fat flap positioned into the bag.

**Figure 9:**
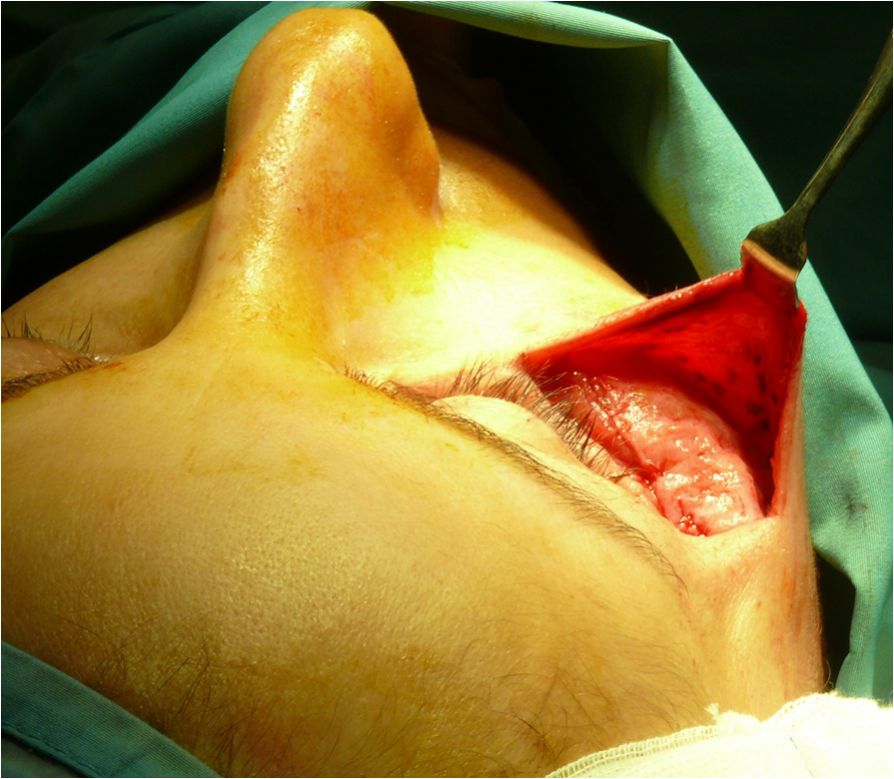
Dermal-fat flap filled into the bag.

The collected tissue, slightly oversized than the surrounding area, was grafted in a skin bag. The bag was detached around the right zygomatic region affected by tissue loss; we used vascularization of the orbicularis oculi muscle to perform the graft (Figures 5, 6, 7, 8, 9).

A small graft was also placed in the right superior palpebral sulcus. Besides, blepharoplasty of the left upper eyelid was also performed; in this way, we corrected the palpebral asymmetry that the patient complained.

## Results

**Figure 10:**
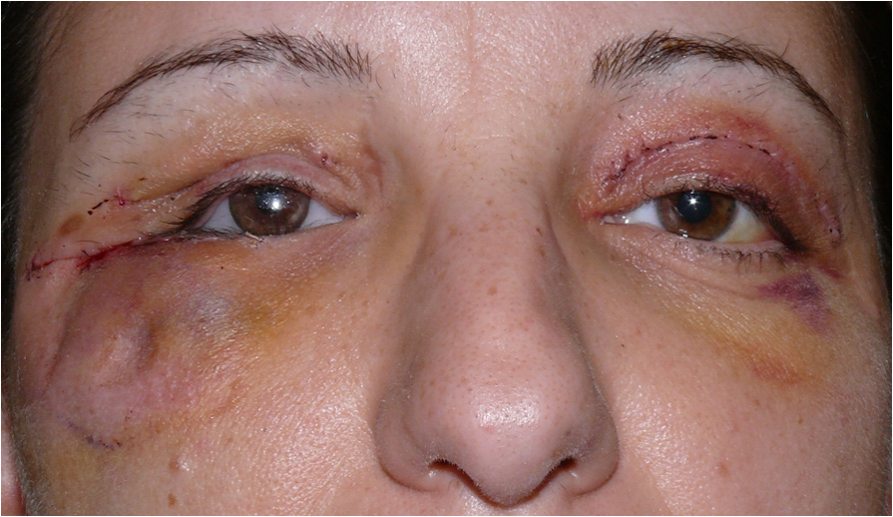
Post-operative picture.

The surgical outcome, 48 hours after the surgery, revealed that the right zygomatic region had returned to its proper anatomical shape, although there were still signs of postoperative edema (Figure 10).

**Figure 11:**
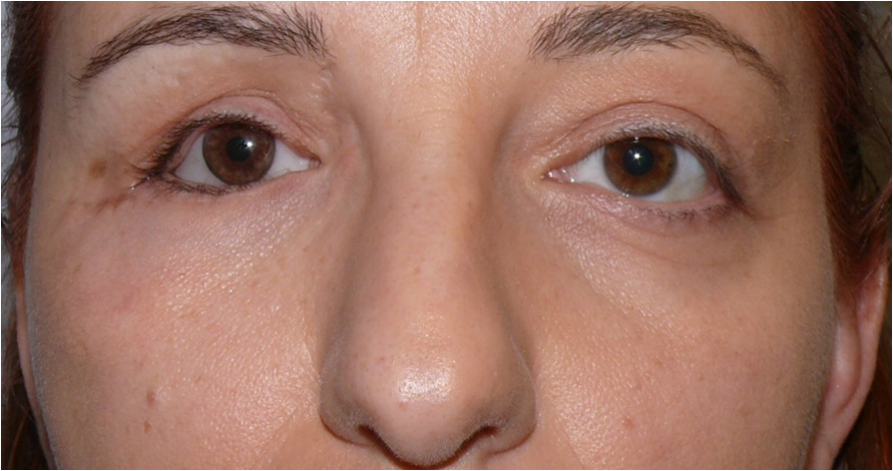
One month follow-up picture.

**Figure 12:**
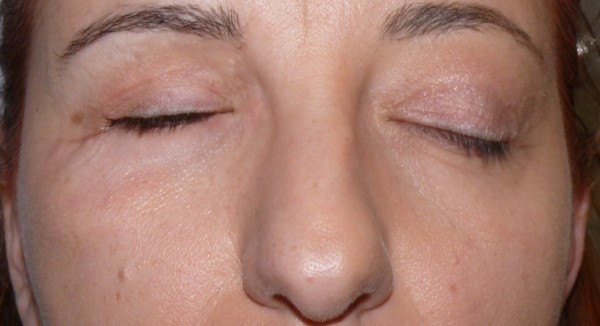
One month follow-up picture.

The tissue graft adhered properly, and there were no evident signs of necrosis or superinfections. The 1 month after follow-up showed that the palpebral areas appeared symmetric and well-proportioned, with patient satisfaction and with no signs of necrosis or superinfections (Figures 11, 12).

## Discussion

The treatment of tissue loss with grafting of autologous adipose tissue in the aesthetic areas is not a widely used technique in plastic and reconstructive surgery; [1,2] as a matter of fact, general literature reports conflicting studies on the use of this procedure for tissue replacement [8-18].

Very damaged tissues, like those exposed to radiation therapy, are generally not suitable for grafting [18,19] of adipose tissue, presumably due to compromised vascularization of the receiving bed caused by prolonged release of cytokines (such as Transforming Growth Factors), which in turn cause fibrosis and angiogenesis with neoformations of the aberrant vascular tissue [10,16].

However, other studies have achieved good results with grafting of autologous adipose tissue in areas previously exposed to radiation therapy, although they reported that resorption was slightly superior to normal tissues [1]. In order to reduce the aesthetic impact resulting from tissue resorption, it is advisable to plan a 20–50% hypercorrection of the grafted tissue [7].

Particular attention should be paid to hemostasis to prevent clot formation at the receiving site; this allows proper vascular anastomosis between the graft and the receiving bed [8].

The orbit has a relatively high fat content, usually in the periorbital region, and is a richly vascularized area; experimental studies have shown how grafting of adipose tissue should be performed where it is particularly present under physiological conditions [1,2,8,12,13]. Besides, it was also demonstrated that graft survival is higher in case of muscle placement [14-21].

## Conclusions

In the described case, we achieved a technically and aesthetically satisfying result despite the patient's medical history involving several perplexities about the use of autologous dermal-fat tissues, because of prior radiation therapy exposure. The clinical case shows that even a region exposed to radiation therapy can be a valid receiving bed for dermal-fat grafting; it is necessary to evaluate the anatomical characteristics of the surgical site to verify vascularization and local compatibility to the presence of adipose tissue in situ. A good hemostasis makes graft vascularization more predictable, thanks to the increased presence of vascular anastomosis between the receiving bed and the graft. One should consider the possibility to graft an oversized dermal-fat flap compared to the volume of the region in order to minimize the aesthetic impact of the adipose tissue, which tends to get reabsorbed over time.

## Consent statement

Written informed consent was obtained from the patient for publication of this case report and accompanying images. A copy of the written consent is available for review by the Editor-in-Chief of this journal.

## Competing interests

All authors disclose any financial and personal relationships with other people or organizations that could inappropriately influence (bias) their work.

## Authors’ contributions

FI and MT equally contributed to this paper: they drafted the manuscript and participated to the analysis of the literature, in the light of the aim of the work. AP, MG and ADI participated in the data collection and analysis, and they revised the literature sources. AMI, GD and MM were involved in the follow-up and in the related data analysis. FMA and LP have performed the surgical cases and optimized the surgical protocol in the light of the state of the art reported in the most recent literature sources. All authors read and approved the final manuscript.
